# Systematic Evaluation of Whole-Genome Sequencing Based Prediction of Antimicrobial Resistance in *Campylobacter jejuni* and *C. coli*

**DOI:** 10.3389/fmicb.2021.776967

**Published:** 2021-11-16

**Authors:** Lisa M. Hodges, Eduardo N. Taboada, Adam Koziol, Steven Mutschall, Burton W. Blais, G. Douglas Inglis, Daniel Leclair, Catherine D. Carrillo

**Affiliations:** ^1^Canadian Food Inspection Agency, Dartmouth, NS, Canada; ^2^Public Health Agency of Canada, Winnipeg, MB, Canada; ^3^Canadian Food Inspection Agency, Ottawa, ON, Canada; ^4^Canadian Food Inspection Agency, Lethbridge, AB, Canada; ^5^Agriculture and Agri-Food Canada, Lethbridge, AB, Canada; ^6^Environment and Climate Change Canada, Ottawa, ON, Canada

**Keywords:** *Campylobacter*, antimicrobial resistance, whole-genome sequence (WGS), bioinformatic tools, AMR surveillance

## Abstract

The increasing prevalence of antimicrobial resistance (AMR) in *Campylobacter* spp. is a global concern. This study evaluated the use of whole-genome sequencing (WGS) to predict AMR in *Campylobact*er *jejuni* and *C. coli*. A panel of 271 isolates recovered from Canadian poultry was used to compare AMR genotype to antimicrobial susceptibility testing (AST) results (azithromycin, ciprofloxacin, erythromycin, gentamicin, tetracycline, florfenicol, nalidixic acid, telithromycin, and clindamycin). The presence of antibiotic resistance genes (ARGs) was determined for each isolate using five computational approaches to evaluate the effect of: ARG screening software, input data (i.e., raw reads, draft genome assemblies), genome coverage and genome assembly software. Overall, concordance between the genotype and phenotype was influenced by the computational pipelines, level of genome coverage and the type of ARG but not by input data. For example, three of the pipelines showed a 99% agreement between detection of a *tet(O)* gene and tetracycline resistance, whereas agreement between the detection of *tet(O)* and TET resistance was 98 and 93% for two pipelines. Overall, higher levels of genome coverage were needed to reliably detect some ARGs; for example, at 15X coverage a *tet(O*) gene was detected in >70% of the genomes, compared to <60% of the genomes for *bla(OXA)*. No genes associated with florfenicol or gentamicin resistance were found in the set of strains included in this study, consistent with AST results. Macrolide and fluoroquinolone resistance was associated 100% with mutations in the 23S rRNA (A2075G) and gyrA (T86I) genes, respectively. A lower association between a A2075G 23S rRNA gene mutation and resistance to clindamycin and telithromycin (92.8 and 78.6%, respectively) was found. While WGS is an effective approach to predicting AMR in *Campylobacter*, this study demonstrated the impact that computational pipelines, genome coverage and the genes can have on the reliable identification of an AMR genotype.

## Introduction

Antimicrobial resistance (AMR) is an increasing global concern to public health, as resistance to common antimicrobials is making it more difficult or impossible to effectively treat human infectious diseases (World Health Organization, 2014). Development of AMR is a natural process in bacteria; occurring through spontaneous mutations in chromosomal genes or by horizontal transfer of genes from one bacteria to another. Acquisition of antibiotic resistance through both point mutations and transfer of antibiotic resistance genes (ARGs) can result in strains with multidrug resistances, leading to very limited treatment options. The emergence or increased prevalence of AMR to critically important antibiotics for human health in the bacterial population has been associated with the unnecessary use of antimicrobial drugs (e.g., over-prescribing in human and animal health, use of antimicrobials for growth-promotion or prophylaxis in animal health) (Inglis et al., 2005; Government of Canada, 2018). In recent years, the need to address increasing AMR in bacterial pathogens has become a global priority (World Health Organization, 2017a,b; Haldenby et al., 2020). This has led to increased surveillance and a reduction in the use of antibiotics, particularly those highly important to human health.

*Campylobacter* spp. are currently the leading cause of food-borne bacterial illnesses in Canada (Government of Canada, 2020b), the United States (Tack et al., 2019), Europe (European Centre for Disease Prevention and Control, 2019), and in many other countries worldwide (Kaakoush et al., 2015). In general, *Campylobacter jejuni* is responsible for >90% of reported cases of campylobacteriosis, followed by *C. coli* (Public Health Agency of Canada, 2018), and together they can represent >99% of the reported illnesses. Campylobacteriosis is typically self-limiting and often characterized by diarrhea, fever and abdominal pain (Skirrow, 1990). In some cases, however, infections by *C. jejuni* can be severe, leading to hospitalization, serious post-infection sequelae such as Guillain-Barré Syndrome (GBS), which results in limb weakness and, in rare cases, total paralysis and death (Skirrow, 1990; Fitzgerald et al., 2021). Antibiotic treatment is usually only given to patients exhibiting severe forms of the disease, or to high-risk individuals (e.g., neonates, pregnant women, and immunocompromised individuals) (Yang et al., 2019). For *Campylobacter* spp., there are increasing rates of fluoroquinolone resistance, specifically, ciprofloxacin resistance being observed in many countries throughout the world (World Health Organization, 2017a,b), including Canada (Inglis et al., 2021). In Canada, ciprofloxacin is classified as an antibiotic of “very high importance” by Health Canada, and it is one of the most prescribed antibiotics for the treatment of campylobacteriosis (Agunos et al., 2013; Inglis et al., 2020). The reason for an increasing prevalence of fluoroquinolone resistance is not known; however, with more extensive surveillance of AMR in *Campylobacter*, potential routes of transmission could become more apparent [reviewed in Sproston et al. (2018)].

Traditional methods for identifying AMR in bacteria include antimicrobial susceptibility testing (AST) via disk diffusion, *E*-test and broth/agar dilution assays. However, as these methods are often time-consuming and laborious, it can be difficult to undertake large scale studies and surveillance programs (Azrad et al., 2018). A relatively recent alternative to these methods is to use whole-genome sequencing (WGS) to screen for the presence of known genetic markers of AMR (Zhao et al., 2016; Whitehouse et al., 2018; Feldgarden et al., 2019; Marotta et al., 2019). Decreasing costs for WGS has led to widespread use of this technology for surveillance and routine testing, in addition to outbreak investigations, as the need for multiple tests can be eliminated when results are inferred from WGS data (e.g., AMR, isolate subtyping, virulence, etc.) (Carrillo et al., 2019). Although the effectiveness of this approach has been previously demonstrated (Page et al., 2015; Zhao et al., 2016; Whitehouse et al., 2018; Feldgarden et al., 2019; Elhadidy et al., 2020; Painset et al., 2020; Rokney et al., 2020; Dahl et al., 2021), to our knowledge, this is the first study that also investigated the impact of bioinformatics tools (e.g., ARG screening and genome assembly software) and level of genome coverage on the ability to predict AMR in *Campylobacter* spp. The objectives of this study were to: (1) compare the WGS-based AMR predictions for *C. jejuni* and *C. coli* isolates to the antimicrobial susceptibility profiles and (2) evaluate how the accuracy of the genotype identification is affected by the choice of computational pipelines and databases used for ARG detection.

## Materials and Methods

### Isolate Collection

All *Campylobacter jejuni* and *C. coli* isolates used in this study were collected between December 2012 and December 2013 as part of the National Microbiological Baseline Study (MBS) conducted by the Canadian Food Inspection Agency in collaboration with industry and government partners (Canadian Food Inspection Agency, 2016). All isolates used in the study were recovered from both federally registered chicken establishments and retail outlets. Antimicrobial susceptibility testing (AST) was conducted as described in below. A subset of 271 isolates (229 *C. jejuni* and 42 *C. coli*) was selected for this study (Supplementary Table 1). The panel consisted of 109 isolates that were phenotypically sensitive to azithromycin (AZM), ciprofloxacin (CIP), erythromycin (ERY), gentamicin (GEN), tetracycline (TET), florfenicol (FLR), nalidixic acid (NAL), telithromycin (TEL), and clindamycin (CLI) and 162 isolates that were resistant to at least one of these antibiotics. Although none of the isolates used in this panel were phenotypically resistant to telithromycin, florfenicol, or gentamicin, the panel did include 37 isolates with an intermediate level of resistance to telithromycin (Supplementary Table 1).

### Antimicrobial Susceptibility Testing

In the case of disagreements between the AST profile and the WGS-derived profile, the AST profile of the isolate was verified as described in Dramé et al. (2020). Briefly, the minimum inhibitory concentration (MIC) for each of the nine antibiotics was determined by the broth dilution method in a microtitre plate format (Public Health Agency of Canada, 2015) and antibiotic susceptibility breakpoints were as described in Dramé et al. (2020) (Supplementary Table 1).

The AST was verified for 13 isolates by broth dilution, based on the methods described by the Public Health Agency of Canada (2015) and National Antimicrobial Resistance Monitoring System (2016) (Supplementary Table 2). Briefly, isolates were streaked for purity on Brucella agar from frozen stocks stored in Brucella broth (Oxoid) with 20% glycerol at −80°C. Isolates were cultured on Mueller Hinton agar (BD Difco) with 5% sheep blood (Cedarlane) at 42°C for 24 h in a microaerobic atmosphere. Cell were harvested from agar plates and cell density was to adjusted to a 0.5 McFarland standard in cation-adjusted Mueller Hinton broth with TES buffer (CAMHB+TES) (Thermo Scientific) using a DensiCHEK Plus instrument (bioMérieux). The cell suspension was then diluted 1:100 in CAMHB+TES with sheep blood (Thermo Scientific) and used as the inoculum for a broth dilution assay. The range of antibiotic concentration tested were as described by Public Health Agency of Canada (2015). Cultures were incubated in the microaerobic atmosphere at 42°C for 24 h. The minimum inhibitory concentration (MIC) was the lowest concentration at which no growth occurred, break-points for each antimicrobial were as described by Public Health Agency of Canada (2015) (Supplementary Table 1).

### Whole-Genome Sequencing and Genome Assembly

Isolates were grown from frozen glycerol stocks by streaking for single colonies on 5% horse blood agar. Cultures were incubated for 24 to 48 h at 42°C in microaerobic conditions. DNA was extracted from single colonies using the EZ1 DNA tissue kit (Qiagen) according to the manufacturer’s instructions. Genomic DNA was quantified using the Quant-it High-Sensitivity DNA Assay Kit (Life Technologies Inc., Burlington, ON, United States) and DNA libraries were prepared from 1 ng of genomic DNA with Illumina Nextera XT DNA sample preparation kit (Illumina, Inc., San Diego, CA, United States) according to manufacturers’ instructions. Whole-genome sequencing was performed on the Illumina MiSeq platform (Illumina Inc.), using a 600 cycle MiSeq reagent kit (v3) generating 2 × 300 bp paired-end reads.

Basic metrics of FASTQ-formatted sequencing reads were determined with FastQC version 0.11.6 (Andrews, 2010). Contamination was detected in sequencing reads using ConFindr (Low et al., 2019). Quality and adapter trimming of reads was performed with the BBduk script from the BBTools suite version 37.78 (Bushnell, 2014) with the following parameters: trim quality of 20, and remove reads below 50 bp long. Error correction was performed using tadpole version 37.78 (Bushnell, 2014) in “correct” mode with default parameters. Trimmed and corrected reads were assembled using SKESA v2.3.0 (Souvorov et al., 2018) and SPAdes v3.13.0 (Bankevich et al., 2012) with shovill 1.0.4^1^. Pilon version 1.2.2 (Walker et al., 2014) was used to automatically improve assembly, and assembly metrics were calculated with a custom Python script^2^, and Qualimap version 2.2.2 (Okonechnikov et al., 2016). Contigs shorter than 500 bp were removed from assemblies. Sequencing was repeated for strains with poor assembly metrics (e.g., low N50, coverage) or when contamination was detected.

### Nucleotide Sequence Accession Numbers

Raw data have been deposited at DDBJ/EMBL/GenBank under BioProject PRJNA600010. The accession numbers and strain descriptions are listed in the Supplementary Table 1.

### Identification of Antimicrobial Resistance Markers in Raw Reads and Assembled Genomes

Identification of antibiotic resistance genes (ARGs) in the raw reads and draft genome assemblies (contig files) of each isolate was completed using the bioinformatics tools and databases listed in Table 1. Genes were deemed to be present based on a minimum sequence identity of 90%. Currently, PointFinder is unable to detect the *gyrA* gene of *C. coli* and at the time analysis the program was unable to reliably detect mutations in the 23S rRNA gene. As such, known point mutations in these two genes were screened for manually. Point mutations conferring AMR in the *gyr*A and the 23S rRNA genes were identified as follows. The *gyr*A and 23S rRNA gene sequences were obtained from the assemblies using GeneSeekr^3^ or from the raw reads using Sipprverse (Lambert et al., 2015; Table 1). Reference sequences for the target genes were from *C. jejuni* NCTC 11168 (accession number AL111168) and *C. coli* RM 4661 (accession number CP007181). Query gene sequences were identified by GeneSeekr through BLASTn (Altschul et al., 1990) sequence comparisons against the reference genes, and enabling the “–fasta_output” option. Sipprverse mapping of raw FASTQ reads to reference gene sequences with a kmer size of 11 and soft-clips enabled, allowed the extraction of the gene sequences. Sipprverse read-baiting was performed with BBduk version 37.78 (Bushnell, 2014). FASTQ reads were mapped to reference files using BOWTIE2 version 2.3.5 (Langmead and Salzberg, 2012), with “–local” alignment, “–very-sensitive-local” scoring, and “–all” alignments reported. BAM files were indexed and sorted with samtools version 1.9 (Li et al., 2009). Alignments were parsed from sorted BAM files using pysam version 0.15.2^4^. Sequence alignments of reference and query genes were done using Geneious Prime (version 2019.2) with nucleotide position numbering of the 23S rRNA gene based on the *C. jejuni* NCTC 11168 reference sequence. Mutations in the *gyrA* gene (amino acid position 86, Thr to Ile) which confer resistance to quinolone and the 23S rRNA gene (positions 2074, A to T, and 2075, A to G), which confer resistance to macrolides/lincosamides/ketolides, were identified.

**TABLE 1:** Bioinformatics tools used to identify antimicrobial resistance genes from assemblies and WGS raw reads.

**Format**	**Tools^a^**	**Assembler**	**Database**
Raw Reads	Sipprverse	N/A	ResFinder^b^
	KMA	N/A	NCBI-AMR^c^
	SRST2	N/A	NCBI-AMR
Assemblies	GeneSeekr	SKESA, SPAdes	ResFinder
	StarAMR^d^	SKESA	ResFinder

*^a^Sipprverse v.0.2.46, KMA v 1.3.3, SRST2 v 0.2.0, GeneSeekr v 0.4.1, and starAMR 0.7.2.*

*^b^Zankari et al. (2012), database version 2021-09-23.*

*^c^NCBI Bacterial Antimicrobial Reference Gene Database (Bioproject: PRJNA313047), version 2021-08-11.1.*

*^d^https://github.com/phac-nml/staramr.*

For isolates with an identified 23S rRNA gene mutation (*n* = 42), sequences of *cmeR*, *cmeABC* operon, L4 (*rspD*), and L22 (*rspV*) were investigated for potential mutations relative to reference sequences from *C. jejuni* and *C. coli*. In addition, the presence of base substitutions (relative to *C. jejuni* NCTC 11168) in the CmeR binding site identified by Lin et al. (2005) were also investigated. Sequences for the genes of interest, including the *tet(O)* gene for isolates carrying the gene, were obtained using Sipprverse and aligned against reference sequences using Geneious (as described above). Isolates carrying a 23S rRNA gene mutation were screened for additional AMR markers using the Comprehensive Antibiotic Resistance Database (CARD) (Alcock et al., 2020).

To evaluate the accuracy of using WGS data to predict AMR in *Campylobacter* the following calculations were made using the phenotype profile as the reference method; overall association ([(a+d)/(a+b+c+d)]x100)), sensitivity ([a/(a+c)] × 100), specificity ([d/(b+d)] × 100), positive predictive value (PPV) ([a/(a+b)] × 100), and negative predictive value (NPV) ([d/(c+d)] × 100), where a = true positives, b = false positives, c = false negatives, and d = true negatives.

### Estimation of the Minimum Read Coverage Required for Detection of ARG

An estimate of the average genome coverage needed to reliably detect an ARG was determined by screening at increasing levels of read coverage. For each isolate, the raw reads were randomly sampled to genome coverage levels of 1, 2.5, 5, 10, 15, 20, 25, and 30X, using the reformat.sh script (version 37.61) provided with the BBMap suite (Bushnell, 2014). Each set of subsampled raw reads was then screened for ARGs using sipprverse (v 0.0.76) with the ResFinder database (2019-04-26), and SRST2 (v 0.2.0) (Inouye et al., 2014), and KMA (v 1.3.3) (Clausen et al., 2018) with the NCBI-AMR database (2019-04-29.1) (Table 1). For all three tools, default settings were used, with a minimum gene coverage was set to 90%. For each isolate, at each genome coverage level, the subsampling and ARG screening was repeated 100X. The expected ARG profile of each isolate was determined by running each of the tools against the raw reads without subsampling. A false positive result was defined as an ARG detected in the subsampled raw reads but not detected using all available raw reads.

## Results

### Concordance Between AST and WGS-Based Predictions

Overall, there was a high concordance between the phenotypic susceptibility and the corresponding genotype (96.3–100%) (Table 2). Of the 271 isolates tested, discrepancies were observed in 13 isolates which were retested using the broth dilution method (Supplementary Table 2). Most of the discrepancies were due to discordance between the CLI and/or TEL genotype and the reported phenotype, i.e., presence or absence of the A2075G 23S rRNA gene mutation but lack of the expected susceptibility profile (Supplementary Table 2). Repeat testing led to the amendment of AST profiles for three of the isolates. Isolates CJ-MBS3796A and CC-MBS0767R were amended as being susceptible to CLI and CJ-MBS3139A was amended to being susceptible to AZM, ERY, TEL, and CLI (Supplementary Table 2). The amended phenotypes were used in subsequent analyses. The remaining isolates which were phenotypically susceptible to CLI and/or TEL but carried the A2075G 23S rRNA gene mutation, were confirmed as being susceptible to the antibiotic.

**TABLE 2:** Concordance of *C. jejuni* and *C. coli* resistance phenotypes to resistance genotypes predicted using GeneSeekr and manual alignment.

**Antibiotic**	**Phenotypic susceptibility^a^**	**Presence of AMR marker (Present/absent)**	**AMR marker identified**	**Overall association**	**Sensitivity**	**Specificity**	**Positive Predictive value**	**Negative Predictive value**
AZM, ERY	R = 42	42/0	23S rRNA A2075G	100%	100%	100%	100%	100%
	S = 229	0/229						
CLI	R = 36, IR = 3	36/0	23S rRNA A2075G	98.9%	100%	98.7%	92.9%	100%
	S = 232	3/229						
TEL	IR = 33	33/0	23S rRNA A2075G	96.3%	100%	96.2%	78.6%	100%
	S = 238	9/229						
TET	IR = 1, R = 130	131/0	*tet(O) tet(O/32/O)*	99.6%	100%	99.3%	99.2%	100%
	S = 140	1/139						
CIP, NAL	R = 30	30/0	*gyrA* T86I	100%	100%	100%	100%	100%
	S = 241	0/241						
GEN	R = 0	0	None detected	100%	100%	100%	100%	100%
	S = 271	0/271						
FFN	R = 0	0	None detected	100%	100%	100%	100%	100%
	S = 271	0/271						

*^a^S = susceptible, IR = intermediate resistance, R = resistance.*

There was a 99.6% association between tetracycline resistance and the detection of a *tet(O)* determinant. The only discordance was a single isolate (CC-MBS7487A), in which a *tet(O)* gene was detected by all of the pipelines but the isolate was susceptible to TET (Supplementary Table 2). The sequences of the *tet(O)* gene from this isolate and all other isolates positive for a *tet(O)* gene (*n* = 128) were aligned against a *tet(O)* reference sequence (accession number M18896). The *tet(O)* sequence of CC-MBS7487A was the only sequence to have a substitution at nucleotide position 1609 (G → T) which resulted in a premature stop codon at amino acid residue 537.

Point mutation in the *gyr*A (T86I) or the 23S rRNA (A2075G) gene was found to correspond 100% to phenotypic resistance to quinolones (CIP and NAL) and macrolides (AZI and ERY), respectively (Table 2). In contrast, there was a lower association between a A2075G 23S rRNA gene mutation and increased resistance to TEL and CLI, with an overall 96.3 and 98.9% association between the predicted AMR and its phenotype for TEL and CLI, respectively. For both TEL and CLI, the ability of WGS to accurately predict sensitivity to each antibiotic was 100%, (i.e., isolates that do not carry a A2075G 23S rRNA gene mutation always corresponded to a susceptible phenotype). In contrast, the PPV (i.e., the accuracy of detecting a mutation and the isolate being resistant to either TEL or CLI) was only 92.9 and 78.6% for CLI and TEL, respectively.

No genes currently known to confer resistance to gentamycin or florfenicol were observed in any of the isolates, resulting in a 100% concordance between genotype and phenotype.

### Effect of Bioinformatics Tools, Genome Assembly and Input Data on AMR Predictions

Overall, the screening software used to identify ARGs (Table 1) had the most impact on both the detection of a gene and the type of gene identified, whereas WGS input (i.e., raw reads or draft assemblies) did not appear to affect the genotype. For example, sipprverse, a software which uses raw reads as input, performed similarly to starAMR and GeneSeekr, software which use draft assemblies as input. While all three pipelines detected the same number of tetracycline resistance genes, there were discrepancies in the detection of two beta-lactam resistance genes; two *bla*OXA genes were not detected by GeneSeekr using SKESA assemblies but were detected in the SPAdes assemblies and by Sipprverse. An investigation into the SKESA assemblies of CJ-MBS1118R and CJ-MBS1595R found that the *blaOXA* gene had not assembled (CJ-MBS1118R) or had only partially assembled (CJ-MBS1595R).

In contrast, KMA, which uses raw reads as WGS input, detected the fewest isolates carrying *tet(O)* and *bla*OXA, with 14 fewer *tet(O)* genes detected than the other pipelines. The pipelines also differed in the detection of genes associated with resistance to various aminoglycosides, as KMA and SRST2 were the only pipelines to detect the *sat4* gene while GeneSeekr detected four additional isolates carrying the *aadE* gene (Table 3). The failure to detect a *sat4* gene by sipprverse was due at least in part to the ARG not found in the ResFinder database. The association between phenotypic resistance and the detection of genes for resistance to beta-lactam or aminoglycosides other than gentamycin was not verified in this study. While all of the pipelines detected tetracycline and beta-lactam resistance [i.e., *tet(O)* and *bla*OXA, respectively], the ability to distinguish between alleles also varied among the pipelines (Table 3). For example, not all pipelines reported a predicted class of *bla*OXA, whereas KMA and SRST2 were the only pipelines that did not identify the mosaic allele *tet(O/32/O)*, which was found in four isolates. With the exception of two *bla*OXA genes, assemblies generated using SKESA and SPAdes reported the same genotype when screened with the same software (i.e., GeneSeekr).

**TABLE 3:** A comparison of AMR gene detection pipelines (*n* = 5) using WGS raw reads and/or assemblies (*n* = 271).

**Resistance**		**Sipprverse**	**KMA**	**SRST2**	**StarAMR**	**GeneSeekr**	
		**Raw Reads**	**Raw Reads**	**Raw Reads**	**Assemblies**	**Assemblies**	
					**SPAdes**	**SKESA**	**SPAdes**
**Tetracycline**							
	*tet(O)*	128	114	129	128	128	128
	*tet(O/32/O)*	4	ND^b^	ND	4	4	4
**Beta-lactamase**							
	*blaOXA* ^a^	219	211	218	219	217	219
**Aminoglycoside resistance genes**							
	*aph(3′)-III*	26	26	23	26	26	26
	*aph(3′)-VIIa*	1	1	1	1	1	1
	*aadE* ^c^	5	4	4	4	9	9
	*sat4*	ND	11	11	ND	ND	ND

*^a^Represents all blaOXA genes identified; class of *blaOXA* gene was predicted for some isolates using StarAMR, KMA and SRST2 (include OXA-61, OXA-184, OXA-193, OXA-448, OXA-449, OXA-465, OXA-466, and OXA-489).*

*^b^ND = none detected.*

*^c^Only identified in *C. coli* isolates.*

### Effect of Genome Coverage on AMR Predictions

**FIGURE 1:**
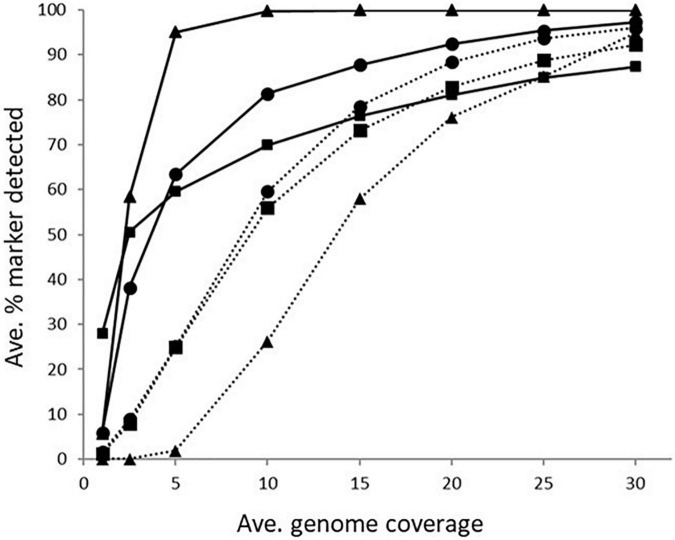
The average percentage of subsampled reads in which *bla*OXA gene (dotted line) and *tet(O)* gene (solid line) was detected in the WGS data at increasing raw read coverage levels. Data points represent the average proportion of subsamples in which a gene was observed that was also observed when each sample was screened using unfiltered raw reads. Symbols represent ^■^KMA, ^•^SRST2, and ^▲^sipprverse.

**FIGURE 2:**
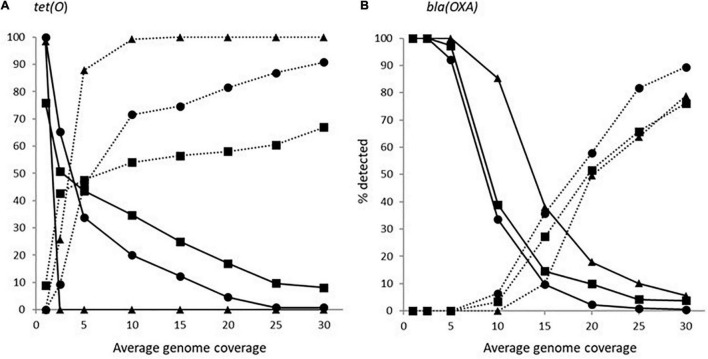
The proportion of *Campylobacter* isolates found to have a *tet(O)* **(panel A)** or a beta-lactamase **(panel B)** marker that had a <50% probability of being detected (solid line) and >90% of being detected (dotted line) using the detection tools KMA (■), SRTS2 (•), and sipprverse (▲) for each level of genome coverage.

The impact of genome coverage on the ability to reliably detect ARGs from raw reads was evaluated using sipprverse, KMA and SRST2. Detection of an ARG was found to be dependent on the screening software, the ARG and the level of genome coverage (Figure 1). Although KMA and SRST2 could detect *bla*OXA genes at lower coverage levels compared to sipprverse, all three pipelines failed to detect the gene 100% of the time for a proportion of isolates using 30X average genome coverage. For example, SRST2 was able to detect the gene in >90% of the subsamples for 89.5% of the isolates (*n* = 194/217) at 30X genome coverage and the gene was missed in only one isolate (0.5%) where it could not be detected in at least 50% of the subsample. In contrast, at 30X genome coverage, the *bla*OXA gene was detected in <50% of the replicates in 3.8% (*n* = 8/217) and 5.5% (*n* = 12/217) of isolates using KMA and sipprverse, respectively (Figure 2).

In comparison to *blaOXA*, a *tet(O)* or *tet(O/32/O)* gene was not only more likely to be detected but it could be detected using significantly lower genome coverage (Figure 1). Of the three software, sipprverse correctly detected a *tet(O)* gene determinant in the most isolates, using the least amount of genome coverage. For example, there was a 95% likelihood of detecting a *tet(O)* gene at only 5X coverage, compared to a likelihood of 60 and 63% using KMA and SRST2, respectively (Figure 1). For example, using KMA false positives were identified in an increasing number of subsamples as genome coverages increased for three isolates, i.e., gene detected in the subsample but not using all available reads. Although the presence of a *tet(O)* gene was not detected using KMA and all available reads, all three isolates were phenotypically tetracycline resistant and a *tet(O)* gene was detected using other pipelines. Using sipprverse, one isolate was reported as a false positive in a proportion of the subsampled reads, identified in up to 20% of the subsamples between one times and 20X coverage. The false positive was not detected by any other pipeline (Table 1) and further investigation using both the assemblies and raw reads suggests the gene is non-functional. The result of a BLAST search of the gene indicates this was likely due to the insertion of a transposon, which corresponded with the isolate being phenotypically susceptible to TET.

## Discussion

In this study, the ability to accurately predict antimicrobial susceptibility using WGS data was evaluated using a panel of 271 *C. jejuni* and *C. coli* isolates recovered from broiler chickens collected in abattoirs and food retail outlets across Canada. While all of the pipelines used in our study were able to identify ARGs associated with a variety of antibiotics (e.g., beta-lactams and tetracycline), the primary focus of this study was on the detection of ARGs and point mutations conferring resistance to the nine antibiotics currently monitored by the national AMR programs in Canada (Government of Canada, 2020a) and in the United States (National Antimicrobial Resistance Monitoring System, 2021).

In addition to the numerous bioinformatics tools that have been developed to screen WGS data for the presence of ARGs (using genome assemblies and/or raw reads), several databases are available that these tools can utilize in order to identify ARGs of interest, leading to a wide range of choices for identifying ARGs in WGS data (Hendriksen et al., 2019; Doyle et al., 2020). Here we have shown that predicting an AMR phenotype from WGS data was dependant on the choice of screening tool, the level of genome coverage and on the specific ARG itself. Of the bioinformatics tools we used, GeneSeekr, sipprverse, and starAMR were found to produce similar results and generated the most accurate profiles with regards to the selected antibiotics. While the completeness of an AMR database used to screen the genome is important for identifying known resistance markers, this was not a significant factor in our study as genes conferring resistance to the priority antibiotics were well represented in both the ResFinder database (Zankari et al., 2012) and the NCBI Bacterial Antimicrobial Resistance Reference Gene Database (Feldgarden et al., 2019). It may, however, explain why KMA and SRST2 detected the presence of an additional *sat4* gene using the more comprehensive NCBI database. Moreover, we observed that software using the same database occasionally produced different results suggesting that the choice of screening tool has a greater impact on results than the choice of database.

Whitehouse et al. (2018) suggested a potential issue with using genome assemblies instead of the raw reads, would be that the software could report the isolate as susceptible when an ARG was split across two contigs. Here, we have shown using two different assembly software, SKESA and SPAdes, that discrepancies between the AMR profiles generated were more likely to be influenced by the screening software and the gene, than by using assemblies generated by using different assembly software. Overall, genotypes generated by GeneSeekr using SKESA and SPAdes assemblies only differed by two *blaOXA* genes. This due to the gene not fully assembling using SKESA, rather than the gene assembling on two contigs. In contrast, it is apparent that the screening tools have considerable impact when using raw reads as the WGS input. For example, of the three pipelines, KMA detected 18 fewer *tet(O)* genes and seven fewer *blaOXA* genes. While the accuracy of detecting aminoglycoside resistance genes or detecting a *blaOXA* gene and predicting the allelic type was not assessed in our study, these results highlight how the choice of screening tool can have a significant impact on the genotype determination.

In our study, we also examined how genome coverage can influence the detection of various ARGs and estimated the minimum level of coverage needed to accurately predict AMR. As expected, the likelihood of detecting an ARG increased with increasing genome coverage. However, each of the three software used (sipprverse, KMA, and SRST2) to map raw reads to the AMR databases was affected differently by the level of coverage in an ARG-specific manner. For example, sipprverse was able to consistently and accurately identify a *tet(O)* marker at very low coverage levels, with an almost 100% detection rate at 5X coverage, whereas using 30X coverage, KMA could not detect the gene in 50% of the subsamples in nearly 10% of isolates. The considerably higher level of genome coverage required by KMA may explain why the pipeline failed to predict tetracycline resistance in 19 isolates that were correctly identified by sipprverse. In contrast, the consistent detection of a *blaOXA* gene required considerably more genome coverage than for a *tet(O)* gene for all the pipelines. In contrast, a similar study performed using KMA with *Salmonella enterica* indicated that ARGs could be accurately identified for more genes with 20 coverage (Cooper et al., 2020). These findings demonstrate that the genetic sequence of a gene also has a significant impact on its detectability, possibly due to the ability of the software algorithms to correctly map the reads to a gene with difficult regions to map (e.g., repetitive regions, multiple copies) and to reach the level of coverage needed to be able to distinguish it from other closely related sequences. This may explain why the use of raw reads as WGS input resulted in considerably more variability in the AMR genotypes between screening software than was observed between software which use draft assemblies as WGS input.

Overall, while it is more cost and time effective to be able to generate profiles with a lower level of coverage, it comes with an increased risk of falsely detecting or not detecting an ARG. For example, in up to 20% of the subsampled reads for one isolate, sipprverse identified a *tet(O)* gene that was non-functional (i.e., insertion of transposon). By ensuring an adequate level of genome coverage and by maintaining stringent cut-off values for detection, the likelihood of a false-positive ARG identification, could be reduced. Due influence that individual genes can play on accurate AMR predictions, our results suggest that the minimum amount of coverage required for these pipelines will largely depend on the organism and the genes of interest.

Consistent with Whitehouse et al. (2018) and Zhao et al. (2016) we observed a strong concordance between the genotype and phenotypic resistance to macrolides (i.e., AZM and ERY), quinolones (i.e., NAL and CIP), and tetracycline. All of the isolates resistant to fluoroquinolone carried a T86I mutation in the *gyrA* gene, consistent with previous studies (Zhao et al., 2016; Whitehouse et al., 2018; Painset et al., 2020), additional substitutions were observed in the gene but were not further investigated. However, a recent study by Webb et al. (2018) identified other base substitutions in the *gyrA* gene which were associated with isolates having a higher level of resistance to ciprofloxacin. In our study, all isolates phenotypically resistant to macrolides were found to have an A2075G mutation in the 23S rRNA gene. In *Campylobacter* spp., the A2075G is the most commonly identified mutation among macrolide-resistant isolates, followed by an A2074T mutation (Bolinger and Kathariou, 2017; Whitehouse et al., 2018). Recently, *Campylobacter* strains carrying an *erm(B)* marker, another macrolide marker, have also been reported although, this is a rare occurrence (Wang et al., 2014). It is often suggested that these two point mutations also confer resistance to lincosamides and ketolides in *Campylobacter* spp.; however, as observed our study and previous studies, the association between these mutations and resistance to lincosamides and ketolides is not conclusive (Whitehouse et al., 2018; Feldgarden et al., 2019). In our study, isolates that did not have an A2075G mutation in the 23S rRNA gene were phenotypically sensitive to both TEL and CLI as was predicted (i.e., negative predictive value), however, the predictability of having a mutation and being resistant to clindamycin or telithromycin was only 92.9 and 78.6%, respectively (i.e., positive predictive value). These rates are similar to those of Whitehouse et al. (2018) who reported rates of 93 and 63% for clindamycin and telithromycin, respectively. Suggested reasons for differences in resistance between strains include insertions, deletions or base substitutions in the L22, L4, *cmeABC* (an efflux pump associated with *C. jejuni*), *cmeR* (regulator of *cmeABC*) and the binding site of CmeR (Lin et al., 2005). However, we were unable to identify mutations in any of these genes or sites which were specific to either resistant or susceptible isolates, as polymorphisms in the sequences were observed in both resistant and susceptible isolates (data not shown). Isolates with an A2075G mutation in the 23S rRNA gene were screened for additional AMR markers using CARD (Alcock et al., 2020), which screens for AMR markers known to cause resistance and genes/markers that may be potential markers of resistance. We did not find an association between any known or suspected AMR marker and resistance to TEL or CLI that could possibly explain why a small number of isolates carry a A2075G mutation in the 23S rRNA gene but are not resistant to either TEL or CLI. The lower accuracy of predicting resistance to lincosamides and ketolides highlights a limitation due to gaps in our understanding of the resistance mechanisms of *Campylobacter* that are reflected in the reference ARG databases, resulting in a risk of either over- or under-estimating the prevalence of AMR in a population.

This study demonstrates the significant impact that the choice of ARG screening software, genome coverage and the gene targets themselves have on the accurate identification of an AMR genotype. Therefore, these factors need to be taken into consideration when screening tools are being developed or are selected for use in a study and/or surveillance pipeline. The genomic data generated for this study can be used as a verified benchmark dataset for assessment of the performance of new computational tools for predicting AMR in *C. jejuni* and *C. coli*. In fact, the genotypes generated by the computational pipelines were in some cases more accurate than those generated by phenotypic susceptibility testing. While the screening tools influence our ability to detect known AMR markers, the accuracy of a genotype is also limited by the comprehensiveness of the reference database. It is important that we continue to study how *Campylobacter* gains resistance to antimicrobials and to understand the mechanisms for AMR in *Campylobacter* spp.

## Data Availability Statement

Raw data have been deposited at https://www.ncbi.nlm.nih.gov/bioproject/PRJNA600010. The accession numbers and strain descriptions are listed in the Supplementary Table 1.

## Author Contributions

LH and CC were responsible for conception and design. LH was responsible for data analysis and preparation of the manuscript. ET, DI, and SM were responsible for all sequencing related to this project, using funding acquired by ET and DI. AK was responsible for bioinformatic support. DL provided all isolates and associated metadata used in this study. CC and BB was responsible for overseeing the bioinformatic support and sequence data. All authors contributed to manuscript revision, read, and approved the submitted version.

## Conflict of Interest

The authors declare that the research was conducted in the absence of any commercial or financial relationships that could be construed as a potential conflict of interest.

## Publisher’s Note

All claims expressed in this article are solely those of the authors and do not necessarily represent those of their affiliated organizations, or those of the publisher, the editors and the reviewers. Any product that may be evaluated in this article, or claim that may be made by its manufacturer, is not guaranteed or endorsed by the publisher.

## Footnotes

1
https://github.com/tseemann/shovill

2
https://github.com/OLC-Bioinformatics/COWBAT

3
https://github.com/OLC-Bioinformatics/GeneSeekr

4
https://github.com/pysam-developers/pysam
