# Supplementary material for: Systematic Evaluation of Whole-Genome Sequencing Based Prediction of Antimicrobial Resistance in Campylobacter jejuni and C. coli
Source: Front Microbiol. 2021 Nov 16;12:776967. doi: 10.3389/fmicb.2021.776967 (PMC8635091; doi:10.3389/fmicb.2021.776967)
Supplement: Supplementary file 2 [file Table_2.DOCX]

**Table S2.** The result of antibiotic susceptibility retesting, using the microtitre dilution method, for *Campylobacter* isolates (n=13) in which a discrepancy was observed between the recorded phenotype and predicted genotype.

| **Antibiotic** | **Isolate ID** | **Species** | **Initial Phenotype** | **Genotype^a^** | **Retested Phenotype** |
| --- | --- | --- | --- | --- | --- |
| AZM/ERY | CJ-MBS0725R | *C. jejuni* | R | S | S |
| TET | CC-MBS7487A | *C. jejuni* | S | R | S |
| TEL | CC-MBS5902A | *C. coli* | S | IR/R | S |
|  | CJ-MBS0725R | *C. jejuni* | R | S | S |
|  | CC-MBS4127A | *C. coli* | S | IR/R | S |
|  | CC-MBS6561A | *C. coli* | S | IR/R | S |
|  | CC-MBS6466A | *C. coli* | S | IR/R | S |
|  | CC-MBS6296A | *C. coli* | S | IR/R | S |
|  | CC-MBS0540R | *C. coli* | S | IR/R | S |
|  | CJ-MBS2360A | *C. jejuni* | S | IR/R | S |
|  | CC-MBS6513A | *C. coli* | S | IR/R | S |
| CLI | CJ-MBS2360A | *C. jejuni* | S | IR/R | S |
|  | CC-MBS6513A | *C. coli* | S | IR/R | S |
|  | CJ-MBS0725R | *C. jejuni* | R | S | S |
|  | CJ-MBS1203A | *C. jejuni* | S | IR/R | S |
|  | CC-MBS3796A | *C. coli* | IR | S | S |
|  | CC-MBS0767R | *C. coli* | IR | S | S |

a. S = susceptible, IR = intermediate resistance, R = resistance
